# UNC93B1 Is Widely Expressed in the Murine CNS and Is Required for Neuroinflammation and Neuronal Injury Induced by MicroRNA *let-7b*

**DOI:** 10.3389/fimmu.2021.715774

**Published:** 2021-09-13

**Authors:** Markus G. Klammer, Omar Dzaye, Thomas Wallach, Christina Krüger, Dorothea Gaessler, Alice Buonfiglioli, Katja Derkow, Helmut Kettenmann, Melanie M. Brinkmann, Seija Lehnardt

**Affiliations:** ^1^Institute of Cell Biology and Neurobiology, Charité – Universitätsmedizin Berlin, Corporate Member of Freie Universität Berlin, Humboldt-Universität zu Berlin, and Berlin Institute of Health, Berlin, Germany; ^2^Department of Neurology, Charité – Universitätsmedizin Berlin, Corporate Member of Freie Universität Berlin, Humboldt-Universität zu Berlin, and Berlin Institute of Health, Berlin, Germany; ^3^Department of Radiology and Neuroradiology, Charité – Universitätsmedizin Berlin, Corporate Member of Freie Universität Berlin, Humboldt-Universität zu Berlin, and Berlin Institute of Health, Berlin, Germany; ^4^Cellular Neuroscience, Max-Delbrück-Center for Molecular Medicine in the Helmholtz Association, Berlin, Germany; ^5^Viral Immune Modulation Research Group, Helmholtz Centre for Infection Research, Braunschweig, Germany; ^6^Institute of Genetics, Technische Universität Braunschweig, Braunschweig, Germany

**Keywords:** UNC93B1, microRNA, *let-7b*, toll-like receptor, neurodegeneration, neuroinflammation, neurons, microglia

## Abstract

The chaperone protein Unc-93 homolog B1 (UNC93B1) regulates internalization, trafficking, and stabilization of nucleic acid-sensing Toll-like receptors (TLR) in peripheral immune cells. We sought to determine UNC93B1 expression and its functional relevance in inflammatory and injurious processes in the central nervous system (CNS). We found that UNC93B1 is expressed in various CNS cells including microglia, astrocytes, oligodendrocytes, and neurons, as assessed by PCR, immunocyto-/histochemistry, and flow cytometry. UNC93B1 expression in the murine brain increased during development. Exposure to the microRNA *let-7b*, a recently discovered endogenous TLR7 activator, but also to TLR3 and TLR4 agonists, led to increased UNC93B1 expression in microglia and neurons. Microglial activation by extracellular *let-7b* required functional UNC93B1, as assessed by TNF ELISA. Neuronal injury induced by extracellular *let-7b* was dependent on UNC93B1, as UNC93B1-deficient neurons were unaffected by the microRNA’s neurotoxicity *in vitro*. Intrathecal application of *let-7b* triggered neurodegeneration in wild-type mice, whereas mice deficient for UNC93B1 were protected against injurious effects on neurons and axons. In summary, our data demonstrate broad UNC93B1 expression in the murine brain and establish this chaperone as a modulator of neuroinflammation and neuronal injury triggered by extracellular microRNA and subsequent induction of TLR signaling.

## Introduction

Toll-like receptors (TLRs) are pattern recognition receptors that mediate innate immune cell signaling in response to both pathogen- and host-derived molecules (1). A subset of endosomal TLRs including TLR3, TLR7, TLR8, and TLR9 recognizes nucleic acids (NA), including double-stranded RNA, single-stranded RNA, and DNA (2–4). In addition, TLR7 was recently discovered to detect extracellular microRNA (miRNA), such as *let-7* (5, 6). UNC93B1 is an endoplasmic reticulum (ER)-resident transmembrane protein that controls TLR trafficking from the ER in peripheral immune cells, such as bone-marrow-derived macrophages and dendritic cells (7, 8). In particular, this chaperone binds to TLR3, TLR7, and TLR9 in the ER and traffics with them to their appropriate intracellular locations where they are subsequently available to sense their cognate ligands (9, 10). TLR trafficking and localization has emerged as a primary mechanism to facilitate self versus non-self discrimination, as well as being crucial for distinct signal transduction in peripheral immune cells. Additionally, UNC93B1 stabilizes TLR proteins and prevents their degradation, functions that are independent of its endosomal trafficking activity (11). However, requirement of UNC93B1 is not restricted to NA-sensing endosomal TLRs, as it is also key to cell-surface expression of the flagellin-sensing TLR5 (12). Other TLRs such as TLR2 and TLR4 are assumed to act independently of UNC93B1 (7, 12). The importance of UNC93B1 in NA recognition and host defense is emphasized by the development of Herpes simplex virus type 1 encephalitis in human patients lacking functional UNC93B1 (13).

Distinct TLRs, expressed in neurons and microglia, the major immune-competent cells in the brain, can elicit central nervous system (CNS) damage (5, 14). In particular, activation of TLR signaling through extracellular microRNA (miRNA) contributes to neuroinflammation and neurodegeneration (5, 15, 16). MiRNAs are ~22-nucleotide noncoding RNAs that, in their conventional form, bind predominantly to the 3′-untranslated regions of mRNAs and regulate their expression at the post-transcriptional level (17). However, a novel role for miRNAs as extracellular signaling molecules has been identified recently, as they can act as endogenous TLR ligands, thereby inducing signaling (5, 18, 19). The highly conserved *let-7b*, one of the most abundant miRNAs in the human brain (20, 21), is released from dying CNS cells and triggers neuroinflammation and neurodegeneration through direct activation of TLR7 (5, 6).

Here, we sought to systematically analyze the expression, regulation, and function of UNC93B1 in the brain in the setting of TLR-mediated neuroinflammation and CNS injury induced by extracellular *let-7b.* We detected the chaperone in various CNS cell populations including neurons and glial cells. Furthermore, UNC93B1 expression in the murine brain increased during development. In neurons and microglia, expression of UNC93B1 was regulated through activation of distinct TLRs, including TLR3, TLR4, and TLR7. Both microglial cytokine release and neuronal injury induced by extracellular *let-7b* required UNC93B1 expression *in vitro*. Likewise, neurodegeneration in the murine cerebral cortex in response to intrathecal *let-*7*b* was dependent on functional UNC93B1. Thus, our data implicate a contribution of UNC93B1 to CNS injury triggered by extracellular miRNA through TLR signaling.

## Materials and Methods

### Mice and Cell Lines

C57BL/6 (wild-type, WT) mice were purchased from Charles River, Sulzbach, Germany. *Unc93b1^-/-^* mice were bred and maintained under specific-pathogen-free conditions at the animal facility of the Helmholtz Centre for Infection Research Braunschweig, Germany, and are described in (22). Animals were maintained and handled in accordance with the guidelines of the committee for animal care, the German Animal Protection Law, and approved by the Regional Office for Health and Social Services in Berlin (Landesamt für Gesundheit und Soziales – LAGeSo, Berlin, Germany).

N1E-115 cells, SH-SY5Y cells, and HMC3 cells were purchased from American Type Culture Collection (Manassas, VA, USA) and maintained in Dulbecco’s Modified Eagle Medium (DMEM) supplemented with 10% heat-inactivated fetal calf serum (FCS, vol/vol) and 1% penicillin/streptomycin. The oligodendroglial precursor cell line Oli-neu was generously provided by Dr. J. Trotter [Institute of Molecular Biology, Johannes Gutenberg-University, Mainz, Germany (23)] and was cultured in DMEM supplemented with 10% fetal bovine serum (FBS) and penicillin/streptomycin (all obtained from Invitrogen, Darmstadt, Germany). Cells were grown at 37°C in humidified air with 5% CO2.

### Primary Cell Cultures of Microglia, Astrocytes, and Neurons

Neonatal primary microglia were isolated from cerebral cortex and midbrain of newborn male and female postnatal (P) 0-3 C57BL/6 and *Unc93b1^-/-^* mice, as described previously (14, 24). In brief, forebrain was freed of blood vessels and meninges. Cortical tissue was trypsinized for 2 min, dissociated with a fire-polished pipette, and washed twice. Mixed glial cells were cultured for 9-12 d in DMEM (Invitrogen, Darmstadt, Germany) supplemented with 10% FCS and 1% penicillin/streptomycin (Gibco, New York, USA), with medium changes every third day. Microglial cells were separated from the underlying astrocytic layer by gentle shaking of the flasks for 1 h at 37°C on a shaker at 100 rpm. Subsequently, microglia were plated. Resulting cell cultures usually contained >95% microglia, as detected by isolectin b4 (IB4) staining (for details see below). Cells were maintained at 37°C in 5% CO_2_ humidified atmosphere.

Cultures of enriched cortical neurons were generated from forebrains of embryonic (E) 17 mice, as described previously (14). In brief, brains were separated from blood vessels, meninges, and cerebellum. Cortical tissue was treated with 500 µl of 2.5% Trypsin (Gibco, New York, USA) for 20 min at 37°C. Trypsin activity was stopped by FBS (Invitrogen, Darmstadt, Germany). Thereafter, cells were washed and incubated with 100 µl DNase (1 mg/ml; Roche Diagnostics, Mannheim, Germany) for 1 min. Subsequently, cells were washed, centrifuged, and plated on poly-D-lysine-coated (Sigma-Aldrich, St. Louis, USA) glass coverslips with Neurobasal Medium (Gibco, New York, USA) supplemented with 1% L-Glutamin, 1% penicillin/streptomycin and 2% B27 supplement (all obtained from Gibco, New York, USA). On the following day, half of media was replaced, and cells were incubated for additional 48 h before starting experiments.

Primary astrocytes were isolated, as previously described (25). In detail, mixed glial cultures were prepared from mouse brains, as described above (see neonatal primary microglia). After separation of microglial cells from the underlying astrocytic layer by gentle shaking of the flasks for 1 h at 37°C on a shaker (100 rpm), astrocytes were trypsinized (2.5%) and subsequently plated.

For microglia/neuron co-culture experiments half of the medium of cultured enriched neurons was replaced by 60,000 microglia in DMEM at day 3 after plating. On the following day, cell cultures were used for experiments.

### Immunocytochemistry and Immunohistochemistry

Immunolabeling was performed as described previously (14). The following primary antibodies were used: anti-NeuN (1:1000 *in vitro*, 1:500 for sections, cat. #ABN78), anti-Neurofilament (1:1000 *in vitro*, 1:500 for sections, clone DA2), anti-microtubule-associated protein 2 (MAP-2, 1:1000, clone AP20), anti-GFAP (1:1000 *in vitro*, 1:500 for sections, clone AB5541), anti-APC (1:300, OP80), anti-active caspase-3 (1:450, clone AB3623, all obtained from Merck Millipore, Burlington, MA, USA), anti-Iba1 (1:1000 *in vitro*, 1:500 for sections; cat. #019-19741, Wako, Neuss, Germany), anti-CD11b (1:500, M1/70, Thermofisher Scientific, cat. #14-0112-82, Waltham, MA, USA), and anti-TLR7 (1:500; Novus Biologicals cat. #NBP2-27332, Centennial, CO, USA. IB4 was obtained from Invitrogen, Darmstadt, Germany. Two rabbit polyclonal antibodies raised against murine UNC93B1 were used in this study: anti-UNC93B1 (#Ab72123, Abcam, Cambridge, UK, used at 1:500 dilution) recognizing the C-terminal region (aa 500-550), and anti-UNC93B1 directed against the N-terminal region (aa 1-59) described in (10), designated anti-UNC93-C and anti-UNC93-N, respectively. Nuclei were stained with 4’,6-diamidino-2-phenylindole (DAPI, 1:10,000, Sigma-Aldrich, Merck KGaA, Darmstadt, Germany). Fluorescence microscopy was performed using an Olympus BX51 microscope (Tokyo, Japan).

### Toxicity Assays

TUNEL staining of CNS cultures was conducted using the In-Situ Cell Detection Kit, TMR Red, following the manufacturer’s recommendations (Roche, Basel, Switzerland). TUNEL staining of brain sections was performed using the ApopTag Fluorescein In-Situ Apoptosis Detection Kit, following the manual’s instructions (Merck Millipore, Darmstadt, Germany). For toxicity studies, the indicated amounts of *let-7b* oligoribonucleotide (5′-UsGsAsGsGsUsAsGsUsAsGsGsUsUsGsUsGsUsGsGsUsU-3′, in which “s” depicts a phosphorothioate linkage; Purimex, Grebenstein, Germany) and other reagents were added to cell cultures for indicated durations. Control cultures were incubated with phosphate-buffered saline. Lipopolysaccharide (LPS; 1 µg/ml, List Biological Laboratories, Campbell, CA, USA) served as positive control for microglia-mediated neuronal injury through TLR4 signaling in microglia/neuron co-cultures. Loxoribine (1 mM, InvivoGen, San Diego, CA, USA) was used as positive control for TLR7-mediated effects. The mutant oligoribonucleotide (5′-UsGsAsGsGsUsAsGsAsAsGsGsAsUsAsUsAsAsGsGsAsU-3′) serving as negative oligoribonucleotide control was synthesized by Purimex, Grebenstein, Germany. For each condition, experiments were performed in duplicates. NeuN-, activated caspase-3-, TUNEL-, and DAPI-positive cells were quantified by analyzing 6 high power fields (at 60x magnification) per coverslip. The viability of control cells was set to 100%. Numbers of NeuN-positive cells observed for each condition were compared with control condition, as indicated, and results were expressed as relative neuronal viability.

### Real-Time PCR

Total RNA was isolated from primary cultured microglia and neurons from WT and UNC93B1-deficient mice, as well as from Oli-neu, N1E-115, SH-SY5Y, and HMC3 cells using InviTrap Spin Universal RNA Mini Kit (Invitek GmbH, Berlin, Germany). RNA quality and yield were determined by NanoDrop ND-1000 (Thermofisher Scientific, Waltham, MA, USA). First, strand cDNA synthesis of RNA was performed using the Superscript II (Invitrogen, Carlsbad, CA, USA) reverse transcriptase according to the manufacturer’s instructions. For mRNA transcription, oligo-dT primers (Invitrogen, Carlsbad, CA, USA) were used. Gene amplification was conducted in duplicates using SYBR Green PCR mix (Applied Biosystems, Foster City, CA, USA) with the following PCR conditions: 95°C for 10 min, 95°C for 15 sec, 60°C for 30 sec, 72°C for 15 sec for 40 cycles using the 7500 Fast Real-Time PCR System (Applied Biosystems, Foster City, CA, USA). Sequences of primers used were specific for the respective candidate molecule (Qiagen, Hilden, Germany): sense 5′GTGCCCTATGCCTACATCCG-3′, anti-sense 5′-CAGCCACCAAGAAGATGTCA-3′ (*Unc93b1*); sense 5′-GCAAGCCAGAGCAGTACTGTG-3′, anti-sense 5′-GCCTCTGTAAGAGATCAGGTAG-3′ (*IRAK1*); sense 5′-CCCTGAAGTACCCCATTGAA-3′, anti-sense 5′-GTGGACAGTGAGGCCAAGAT’-3′ (*β-actin*). 5′-CATCACTGCCACCCAGAAGACTG-3′, anti-sense 5′-ATGCCAGTGAGCTTCCCGTTCAG-3′ (*Gapdh*). Changes in mouse UNC93B1 gene expression were analyzed by the comparative 2(–ΔΔCt) method relative to β-actin or GAPDH gene expression levels, as indicated.

### Intrathecal Injection Into Mice

Intrathecal injection into mice was performed as described previously (5, 26). 10 µg RNA were used for injections, and brains were analyzed 72 h later. After transcardial perfusion with 4% paraformaldehyde (PFA, vol/vol), brains were removed and cryoprotected in 30% sucrose (vol/vol). Cryostat coronal sections (15 µm) were thaw-mounted on coated glass slides. Representative brain sections (level 1: interaural 6.60 mm; level 2: 5.34 mm; level 3: 3.94 mm; level 4: 1.86 mm; level 5: -0.08 mm) were fixed with 4% PFA, washed in PBS, and treated with blocking solution (5% normal goat serum) for 3 h. Sections were then incubated with the respective primary antibody overnight at 4°C. Subsequently, sections were incubated with the relevant secondary antibody (all purchased from Jackson Immuno Research, West Grove, USA) for 1 h at room temperature.

### Quantification of CNS Cells in Brain Sections

Numbers of neurons in the cerebral cortex were assessed by quantifying NeuN-positive cells in 6 fields (at 60x magnification) of the right and left cerebral cortex at level 4 of the 5 representative sections in each brain (see above). The mean was calculated, which is expressed as NeuN-positive cells per mm^2^. For analysis of apoptotic cells, brain sections were stained with TUNEL assay and DAPI. TUNEL-positive nuclei were counted in the cerebral cortex from the representative levels 1 through 5 (see above) after visual verification of apoptotic hallmarks such as shrinkage, blebbing, and fragmentation, and the sum per field was calculated. Each group is displayed with the mean.

### TNF-α Enzyme-Linked Immunosorbent Assay

Primary cultures of microglia were generated from C57BL/6 mice as described above. Microglia were incubated with 5 µg/ml of *let-7b* or mutant oligoribonucleotide for indicated durations, or were exposed to increasing concentrations of *let-7b* or mutant oligoribonucleotide, as indicated, complexed to the transfection agent LyoVec (InvivoGen #LYEC-RNA, San Diego, CA, USA) for 12 h. Conditioned medium was collected and analyzed by enzyme-linked immunosorbent assay with hamster antibody against mouse TNF*-*α (BD Biosciences, Franklin Lakes, NJ, USA) as a capture antibody and biotin-labeled antibody against mouse as secondary antibody (BD Biosciences, Franklin Lakes, NJ, USA).

### Flow Cytometry

Murine microglia, astrocytes, and neurons were isolated as described above. After fixation and permeabilization (Cytofix/Cytoperm Kit, BD Biosciences, Heidelberg, Germany), cells were incubated for 30 min at 4°C with the following antibodies: anti-CD11bPacBlue (M1/70, eFluor 450, cat. #480112-82, Thermofisher Scientific, Waltham, MA, USA), anti-GLAST (ACSA-1)-APC (Miltenyi Biotec, cat. #130-095-814, Bergisch Gladbach, Germany), anti-βIII-Tubulin (clone: TUJ1, BD Biosciences, cat. #560394, Franklin Lakes, NJ, USA), anti-UNC93B1-N (1:500), with secondary PerCP conjugate and their recommended isotype controls (Thermofisher Scientific, Waltham, MA, USA). All antibodies were used at 1:100, unless indicated otherwise. Blocking of Fcγ-receptors (Thermofisher Scientific, Waltham, MA, USA) was conducted before cell surface and intracellular staining. Flow cytometric analysis was performed on FACS Canto II (BD Biosciences, Heidelberg, Germany) and analyzed by FlowJo software (TreeStar, Inc.). UNC93B1 expression was indicated as median fluorescence intensity and presented in histograms.

### RNA-Seq Data Extraction

The following RNA-seq datasets (available at http://www.brainrnaseq.org) were extracted: i) RNA-seq data from various murine embryological (E), postnatal (P), and adult microglia (27, developmental stages indicated in **Supplementary Figure 1**), ii) astrocytes, neurons, oligodendrocyte precursor cells, newly formed oligodendrocytes, myelinating oligodendrocytes, microglia/macrophages, and endothelial cells from the adult mouse cerebral cortex (28), and iii) fetal astrocytes, mature astrocytes, neurons, oligodendrocytes, microglia/macrophages, and endothelial cells purified from the temporal lobe of juvenile, adult or fetal human patients (29). Gene expression levels were expressed as fragments per kilobase per million (FPKM).

### Statistical Analysis

Data are expressed as mean ± SEM or ± SD, as indicated. Statistical differences over all groups were determined by the non-parametric Kruskal-Wallis or one-way ANOVA test, as indicated. Statistical differences within the group and between the groups were determined by Dunn’s multiple comparison post-hoc test, or unpaired Student’s *t*-test, respectively. Statistical differences were considered to be significant when *p* < 0.05.

## Results

### UNC93B1 Is Expressed in Various CNS Cell Populations, Including Neurons and Glial Cells

**Figure 1:**
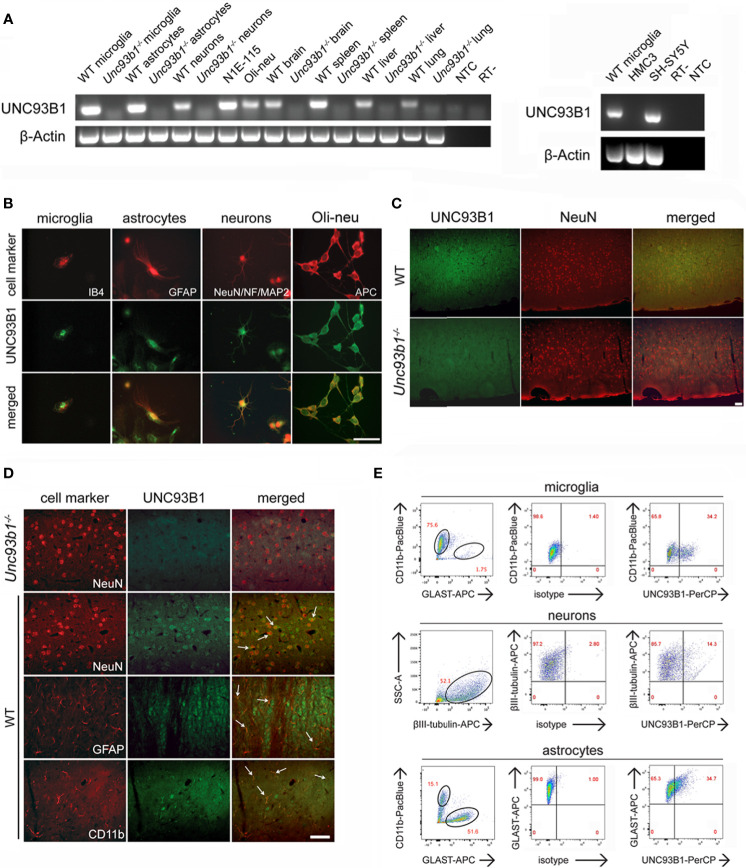
UNC93B1 is expressed in various CNS cell types. **(A)** Total RNA was extracted from purified cortical neurons, astrocytes, and microglia, as well as from whole brain, spleen, liver, and lung, derived from C57BL/6 (wild-type, WT) and *Unc93b1^-/-^* mice. In addition, RNA from N1E-115 cells, Oli-neu cells (left panel), as well as human-derived HMC3 and SH-SY5Y cells (right panel) was isolated. Samples were analyzed by RT-PCR, using primers for *Unc93b1*. β-actin served as loading control. NTC, non-template control; RT-, condition without reverse transcriptase. **(B)** Purified WT cortical neurons, microglia, and astrocytes were immunolabeled with an antibody directed against the C-terminus of UNC93B1 (anti-UNC93B1-C) and co-stained with IB4 to mark microglia, with GFAP antibody to mark astrocytes, and with MAP-2, NeuN, and neurofilament (NF) antibodies to label neurons. Oli-neu cells were immunolabeled with the UNC93B1-C antibody and the oligodendrocyte marker APC. Scale bar, 50 µm. **(C, D)** Brain sections from WT and *Unc93b1^-/-^* mice were immunolabeled using antibodies directed against UNC93B1-C, NeuN, GFAP, and CD11b, the latter serving as markers for neurons, astrocytes, and microglia/brain macrophages, respectively. An overview **(C)** and higher magnification images **(D)** of the cerebral cortex displaying the respective CNS cell populations are shown. Arrows indicate cells co-labeled with UNC93B1 antibody and the respective cell type marker. Scale bar, 50 µm. **(E)** WT brain tissue was dissociated, and cells were stained with both anti-UNC93B1-N antibody, recognizing the N-terminal region of UNC93B1, and different surface markers, as indicated. Microglia were defined as CD11b^+^GLAST^-^, astrocytes as CD11b^-^GLAST^+^, and neurons as CD11b^-^CD45^-^βIII-tubulin^+^ cells. Subsequently, cells were analyzed by flow cytometry. One representative experiment out of 3 with similar results is shown.

We sought to investigate UNC93B1 expression and its functional relevance in the brain. To this end, we first analyzed different enriched CNS cell types such as microglia, astrocytes, and neurons, as well as whole brain tissue derived from C57BL/6 (wild-type, WT) mice regarding *Unc93b1* mRNA expression by end-point PCR. For comparison, tissue from WT spleen, liver, and lung were included in this experimental approach. Respective cells and tissue isolated from *Unc93b1^-/-^* mice served as negative control. In addition, we tested Oli-neu cells representing an oligodendrocyte precursor cell system, and the murine neuroblastoma cell line N1E-115, the latter exclusively containing neuron-derived cells and thus suitable to rule out expression signals due to potentially contaminating non-neuronal cells such as glia, for *Unc93b1* expression (**Figure 1A**). *Unc93b1* RNA was readily detectable in WT whole brain, its expression signal comparable to that in lung and liver tissue, but at lower level compared to spleen tissue. Primary microglia and astrocytes derived from WT mice showed similar *Unc93b1* transcript amounts, while Oli-neu cells and primary WT neurons also displayed a distinct, but comparatively weaker signal (**Figure 1A**). PCR analysis also revealed *Unc93b1* RNA expression in N1E-115 cells, thereby validating UNC93B1 expression in neuronal cells. In contrast to the findings on the different WT-derived tissues and cell types described above, *Unc93b1* RNA was not detected in any cell or tissue sample derived from *Unc93b1^-/-^* mice, confirming the specificity of the expression signals observed in WT samples (**Figure 1A**). *Unc93b1* RNA was readily detectable in the human neuroblastoma cell line SH-SY5Y, while no significant expression was found in the human microglial cell line HMC3 (**Figure 1A**). Analysis of previously published RNA-Seq datasets derived from various murine and human brain cell populations (27–29) identified microglia as the predominant CNS cell type expressing *Unc93b1* transcripts at comparatively high level (**Supplementary Figure 1**). Also, these data indicate a lower *Unc93b1* expression level in human microglia compared to mouse-derived cells. However, although at significantly lower level, all tested CNS cell populations, including neurons, were found to express *Unc93b1* RNA and corroborated our findings on constitutive UNC93B1 expression in the brain assessed by RT-PCR, as described above.

Immunocytochemical analysis of WT cell cultures with an UNC93B1 antibody revealed cytosolic UNC93B1 protein expression in cortical neurons, microglia, and astrocytes. Also, Oli-Neu cells displayed distinct UNC93B1 expression (**Figure 1B**). Next, to analyze UNC93B1 protein expression in the cerebral cortex, brain sections of both WT and *Unc93b1^-/-^* mice were immunolabeled with an UNC93B1 antibody. WT cerebral cortex displayed widespread UNC93B1 expression (**Figure 1C**), and co-immunolabeling with a NeuN antibody identified UNC93B1-positive cells mainly as neurons (**Figures 1C, D**). However, although to a much lesser extent, UNC93B1 expression was also detected in microglia and astrocytes, as assessed by immunolabeling with CD11b and glial fibrillary acidic protein (GFAP) antibodies, respectively (**Figure 1D**). The specificity of the UNC93B1 antibody used for immunohistochemistry was confirmed by labeling brain sections derived from *Unc93b1^-/-^* mice, in which no specific signal was detected after exposure to the UNC93B1 antibody (**Figures 1C, D**). Expression and localization of TLR7, one of the chaperone’s client receptors, in the cerebral cortex did not significantly differ between WT and *Unc93b1^-/-^* mice (**Supplementary Figure 2**).

In order to quantify UNC93B1 protein expression in cortical neurons, microglia, and astrocytes we performed flow cytometry. To this end, whole brain tissue from WT mice was dissociated and labeled with different cell-specific surface markers, as well as an UNC93B1 antibody. Microglia were defined as CD11b^+^GLAST^-^, astrocytes as CD11b^-^GLAST^+^, and neurons as CD11b^-^CD45^-^βIII-tubulin^+^ cells (**Figure 1E**). As indicated in the representative dot plot, 34.2% of microglia, 14.3% of neurons, and 34.7% of astrocytes expressed UNC93B1 (**Figure 1E**). The specificity of the UNC93B1 antibody used for flow cytometry was confirmed by analyzing *Unc93b1^-/-^* astrocytes, in which no UNC93B1 expression was detectable (**Supplementary Figure 3**).

In summary, our expression studies revealed constitutive UNC93B1 expression in various CNS cell populations, including microglia, as well as neurons, astrocytes, and oligodendrocytes.

### UNC93B1 Expression Increases During Brain Development

**Figure 2:**
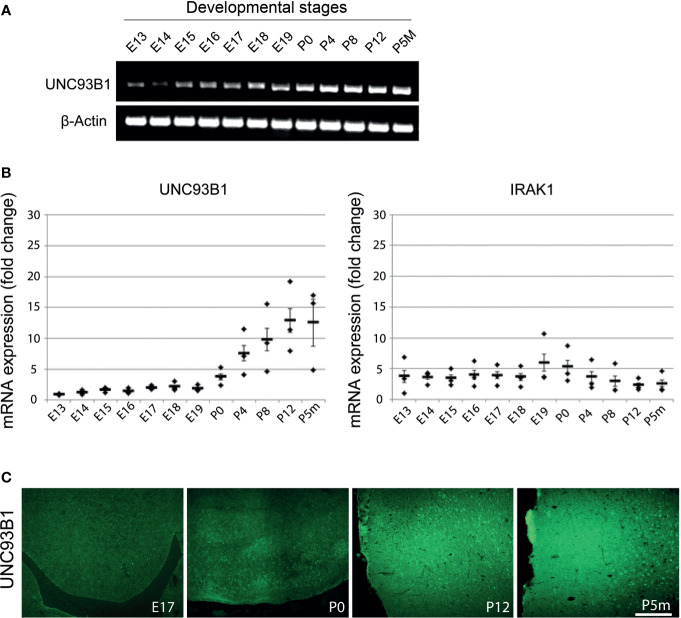
Expression of UNC93B1 in the mouse brain increases during development. Brain homogenates from C57BL/6 mice at various embryonic (E), postnatal (days after birth, P), and adult (age of months, Pm) stages, as indicated, were assayed by endpoint RT-PCR **(A)** and quantitative real-time PCR **(B)** using primers for *Unc93b1* and *IRAK1*. Relative quantification was assessed using the formula 2^–ΔCT^ and by normalizing the amount of the target gene to the housekeeping genes β-actin **(A)** and GAPDH **(B)**. The mean value of E13 was set to 1-fold induction, and mean values of all other developmental stages were related to E13 (*n* = 3). Results are presented as mean ± SD. **(C)** Immunolabeling of the C57BL/6 cerebral cortex at different developmental stages, as indicated, with the UNC93B1-C antibody. Scale bar, 200 µm.

To analyze UNC93B1 expression in the developing mouse brain, brain tissue from C57BL/6 mice at 12 different developmental stages including E13-19, P0, postnatal days P4, P8, and P12, as well as the age of 5 months (P5m) were analyzed by semiquantitative (**Figure 2A**) and quantitative real-time (**Figure 2B**) PCR using primers specific for UNC93B1. For quantitative comparison, interleukin-1 receptor-associated kinase 1 (IRAK1), a component of the canonical TLR downstream signaling pathway (1), was included in this experiment. Relative quantification was assessed using the formula 2^–ΔCT^ and by normalizing the amount of the target gene to the housekeeping gene GAPDH, whose expression levels were not significantly altered during brain development (data not shown). Expression of *Unc93b1* mRNA in the brain steadily increased during the different developmental stages, reaching a peak at P12 (**Figures 2A, B**), whereas *IRAK1* expression did not significantly change during the whole analyzed period (**Figure 2B**). In accordance with our findings on *Unc93b1* mRNA expression in the developing brain, immunohistochemical analysis of the WT neocortex revealed increasing UNC93B1 expression through the different embryonic, postnatal, and adult stages (**Figure 2C**).

### UNC93B1 Expression in Microglia and Neurons Is Regulated by Distinct TLR Activators, Including Extracellular *let-7b* miRNA

**Figure 3:**
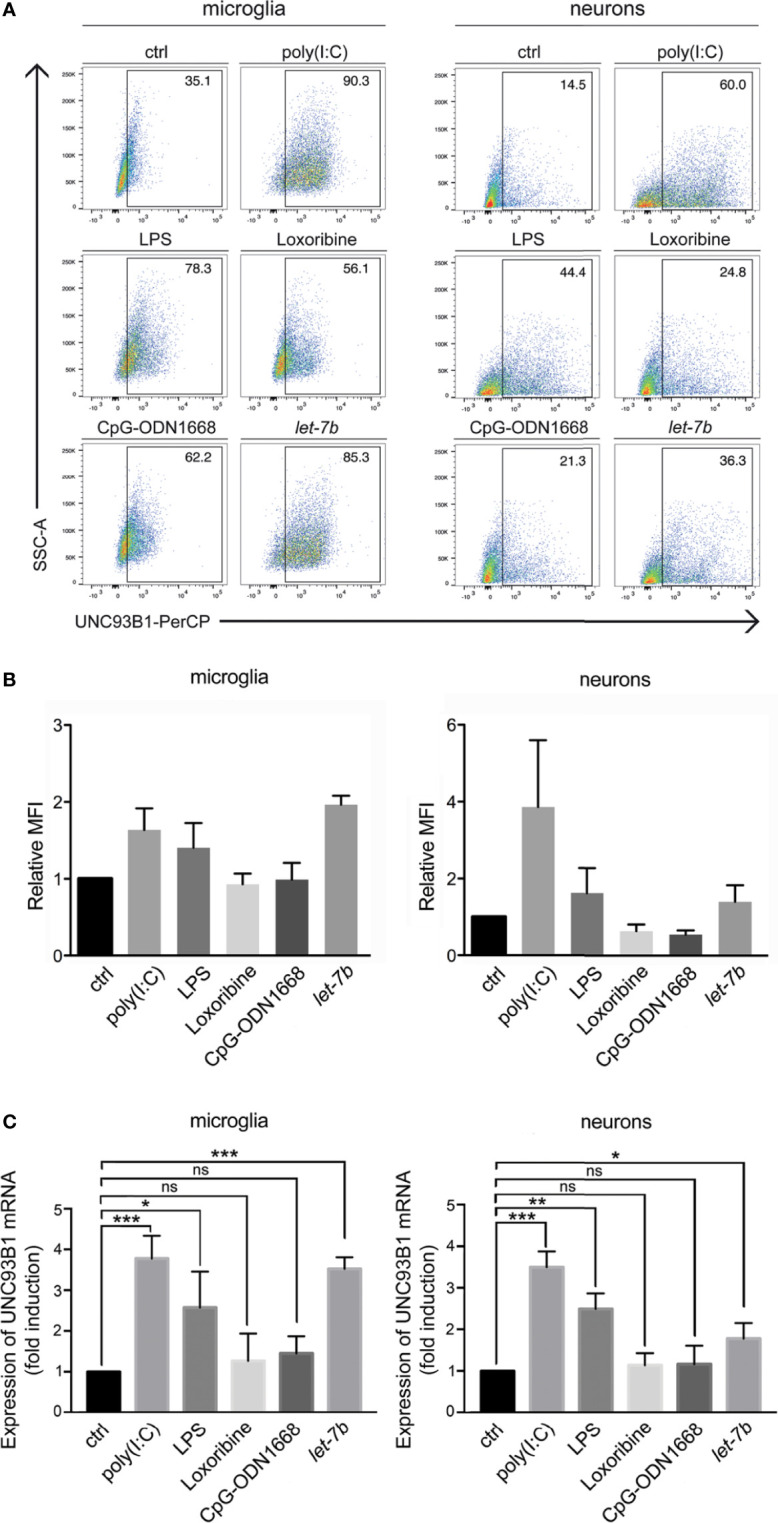
UNC93B1 expression in microglia and neurons is regulated by TLR activation. **(A)** Microglia and neurons isolated from C57BL/6 mice, were exposed to poly(I:C) (150 µg/ml), LPS (1 µg/ml), loxoribine (1 mM), CpG-ODN 1668 (50 µM), or *let-7b* oligoribonucleotide (20 µg/ml) for 6 h Subsequently, cells were dissociated, labeled with both anti-UNC93B1-N and the respective antibodies for cell-specific markers, and were analyzed by flow cytometry. **(B)** Results were expressed as fold change of UNC93B1 PerCP median fluorescence intensity of CD11b^+^ (microglia) and betaIII-tubulin^+^ (neurons) cells (relative MFI; TLR agonist treatment *vs.* control, ctrl; *n* = 3) and are presented as mean ± SEM. **(C)** qPCR analysis of the cell cultures treated as described above using primers against UNC93B1 was performed. β-actin served as loading control. Relative *Unc93b1* mRNA expression levels (TLR agonist treatment *vs.* control, ctrl) are shown (*n* = 3). Results are presented as mean ± SEM. *P* values for relevant groups were determined by Student’s *t* test. p* < 0.05; **p < 0.01; ***p < 0.001; ns, not significant.

While microglia express all TLRs identified to date, neurons express only a few TLRs under certain conditions (30, 31). We sought to determine whether UNC93B1 expression in microglia and neurons is affected by TLR activation. To this end, enriched microglia and neurons derived from WT mice were exposed to the TLR3 agonist poly(I:C), the TLR4 agonist LPS, the TLR7 agonist loxoribine, and to CpG ODN1668, a TLR9 agonist. Also, cells were incubated with the oligoribonucleotide *let-7b*, which was recently identified as an endogenous TLR7 activator (5, 6). After 6 h, cells were dissociated and immunostained with an UNC93B1 antibody and the respective cell type-specific surface markers. Subsequently, cells were analyzed by flow cytometry (**Figure 3A**), and UNC93B1 expression was quantified by assessing median fluorescence intensity (MFI; **Figure 3B**). Poly(I:C) and LPS treatment resulted in increased UNC93B1 expression in both microglia and neurons. Likewise, exposure to extracellular *let-7b* led to an increase in relative UNC93B1 expression in these cells (**Figures 3A, B**). UNC93B1 expression in microglia and neurons treated with loxoribine or CpG ODN1668 was not significantly altered compared to control (**Figures 3A, B**). To determine expression changes of *Unc93b1* mRNA in microglia and neurons exposed to these TLR activators, qPCR with primers against *Unc93b1* was performed (**Figure 3C**). Consistent with our findings on UNC93B1 protein expression, poly(I:C) and LPS treatment resulted in a significant increase in relative *Unc93b1* mRNA expression in both microglia and neurons (microglia: poly(I:C), 3.79 ± 0.32, *p* = 0.001; LPS, 2.59 ± 0.51, *p* = 0.036; neurons: poly(I:C), 3.51 ± 0.22, *p* = 0.0003; LPS 2.5 ± 0.22, *p* = 0.0023; **Figure 3C**). Likewise, *let-7b* miRNA treatment induced a significant increase of relative *Unc93b1* mRNA expression in both cell types (microglia: 3.54 ± 0.16, *p* = 0.0001; neurons: 1.79 ± 0.22, *p* = 0.0215; **Figure 3C**). Exposure to loxoribine and CpG ODN1668 had no effect on *Unc93b1* transcript levels, neither in microglia nor in neurons (microglia: loxoribine, 1.28 ± 0.39, *p* = 0.5154; CpG ODN1668, 1.46 ± 0.24, *p* = 0.131; neurons: loxoribine, 1.15 ± 0.17, *p* = 0.4331; CpG ODN1668, 1.17 ± 0.26, *p* = 0.5444; **Figure 3C**).

Taken together, *let-7b*, poly(I:C), and LPS, established TLR7, TLR3, and TLR4 activators, respectively, induced UNC93B1 expression in microglia and neurons.

### UNC93B1 Is Required for *let-7b*-Induced Neuroinflammation And Neuronal Injury Mediated by Microglia *In Vitro*

**Figure 4:**
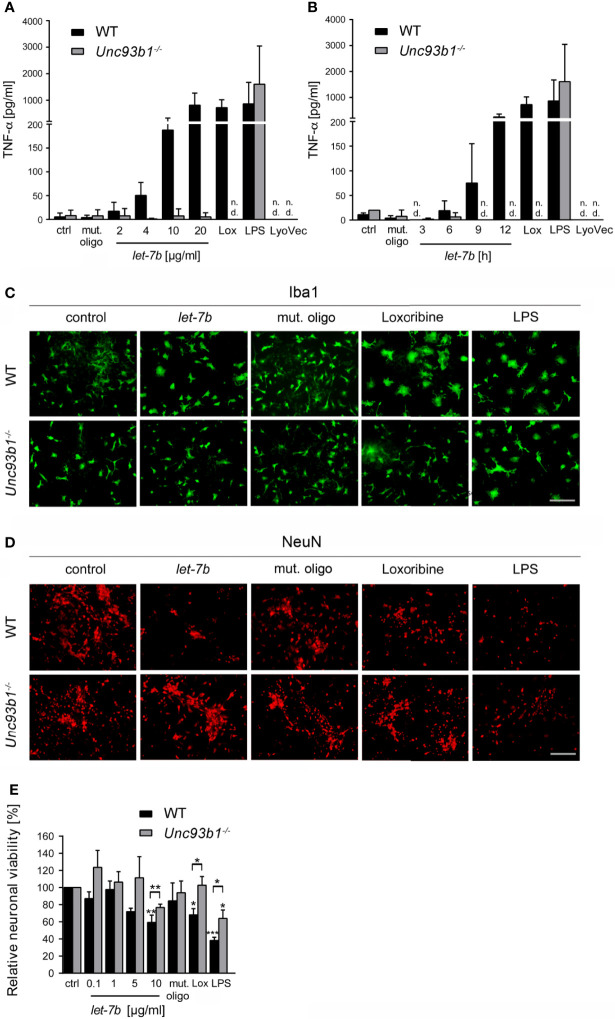
Neuroinflammatory response and neuronal injury in response to extracellular *let-7b* are dependent on UNC93B1 *in vitro*. Microglia derived from C57BL/6 (wild-type, WT) and *Unc93b1^-/-^* mice were incubated with various doses of *let-7b* oligoribonucleotide, as indicated, for 12 h **(A)**, or with 10 µg/ml *let-7b* for various durations, as indicated **(B)**. Mutant oligoribonucleotide (20 µg/ml, 12 h) and the transfection agent LyoVec served as negative control. LPS (100 ng/ml, 12 h) was used as positive control for microglial TNF-α release through TLR4 activation, while loxoribine (1 mM, 12 h) served as control for TLR7-dependent TNF-α release. Subsequently, supernatants were analyzed by TNF-α ELISA. Results are presented as mean ± SD. *n* = 4; n.d., not detectable. **(C–E)** WT neurons co-cultured with either WT or *Unc93b1^-/-^* microglia were treated with various *let-7b* doses (**C, D**: 10 µg/ml for 72 h; **E**: as indicated for 5 d). Mutant oligoribonucleotide (5 µg/ml) served as negative control, LPS (100 ng/ml) served as positive control for microglial activation, and loxoribine (10 mM) served as positive control for TLR7-dependent microglial activation. Subsequently, co-cultures were immunolabeled with Iba1 **(C)** and NeuN **(D)** antibodies to mark microglia and neurons, respectively. Scale bar, 50 µm. **(E)** Quantification of NeuN-positive cells in co-cultures treated as described above. Untreated control was set to 100%. For each condition, experiments were performed in duplicates. At least 3 independent experiments were performed. Data are expressed as mean ± SEM. Kruskal-Wallis test was used to determine global significance over all conditions (*p* = 0.0406 over WT and *p* = 0.0897 over *Unc93b1^-/-^*). *P* values of relevant groups (indicated condition *vs.* unstimulated control, ctrl) were determined by Dunn’s multiple comparison test. *P* values between the respective groups of WT *vs. Unc93b1^-/-^* (in brackets) were assessed by unpaired Student’s *t* test. *p < 0.05; **p < 0.01; ***p < 0.001.

Activation of TLRs in the CNS leads to the release of cytokines and chemokines from microglia, thereby triggering a neuroinflammatory response and subsequent brain tissue injury (32). In particular, TLR7 activation by extracellular miRNA such as *let-7b* contributes to neuroinflammation (5, 6). However, the role of UNC93B1 in this context has not been explored yet. Thus, we analyzed supernatant from WT and *Unc93b1^-/-^* microglia incubated with increasing doses of *let-7b* after 24 h or with 5 µg/ml *let-*7b for various time periods by TNF-α ELISA (**Figures 4A, B**). While loxoribine was used as positive control for TLR7-induced microglial activation, LPS served as positive control for microglial activation through TLR4. A mutant oligoribonucleotide harboring the *let-7b* sequence with reduced GU content (containing six nucleotide exchanges in the central and 3′ regions), which does not activate TLR7 (5), served as negative control in this experimental set-up. WT microglia released TNF-α in response to *let-7b*, and this effect was dose (**Figure 4A**)- and time (**Figure 4B**)-dependent, as expected. *let-7b*-induced TNF-α release from microglia required UNC93B1, as *Unc93b1^-/-^* microglia failed to respond to *let-7b* during the entire time course (**Figures 4A, B**). Likewise, while loxoribine induced TNF-α release from microglia, no such response was detected in cell cultures containing UNC93B1-deficient microglia (**Figures 4A, B**). In contrast and as expected, LPS-induced cytokine release was not dependent on UNC93B1 expression, as both WT and *Unc93b1^-/-^* microglia released similar TNF-α amounts (**Figures 4A, B**). The control mutant oligoribonucleotide did not induce microglial TNF-α release during the whole time period observed, regardless of the genotype, as expected (**Figures 4A, B**).

Activation of microglial TLRs, including TLR7 and TLR4, can contribute to neuronal injury (5, 14, 15). We analyzed the role of UNC93B1 in this context, particularly in the setting of neuronal damage triggered by extracellular *let-7b*. To this end, WT neurons co-cultured with WT microglia or *Unc93b1^-/-^* microglia were incubated with *let-7b* or mutant oligoribonucleotide. LPS served as positive control for neuronal injury through TLR4 in microglia. Subsequently, co-cultures were immunolabeled with Iba-1 (**Figure 4C**) and NeuN (**Figure 4D**) antibodies to mark microglia and neurons, respectively. While immunocytochemistry revealed morphologically transformed microglia in *let-7b*-treated co-cultures, indicating cellular activation and resembling those treated with loxoribine, the morphology of *Unc93b1^-/-^* microglia in response to *let-7b* treatment did not differ from that of unstimulated microglia (**Figure 4C**). Exposure of both co-cultures containing WT or *Unc93b1^-/-^* microglia to LPS led to a cell shape indicating activation, as expected (**Figure 4C**). Quantification of neurons in *let-7b*-treated co-cultures containing WT microglia revealed a dose-dependent decrease in relative neuronal viability, as expected (**Figures 4D, E**). In contrast, co-cultures containing *Unc93b1^-/-^* microglia did not exhibit significant loss of neurons when using *let-7b* doses as high as 10 µg/ml. Numbers of neurons in these co-cultures exposed to *let-7b* did not significantly differ from those in cell cultures exposed to the mutant oligonucleotide (Student’s *t*-test, *p* = 0.1971). Loxoribine induced a reduction in neuronal viability in co-cultures containing WT microglia, but not in those containing UNC93B1-deficient microglia. LPS treatment of both co-cultures containing WT or *Unc93b1^-/-^* microglia led to a reduction in neuronal viability (**Figures 4D, E**).

### Cell-Autonomous Neuronal Injury Induced by Extracellular *let-7b* Is Dependent On UNC93B1 *In Vitro*

**Figure 5:**
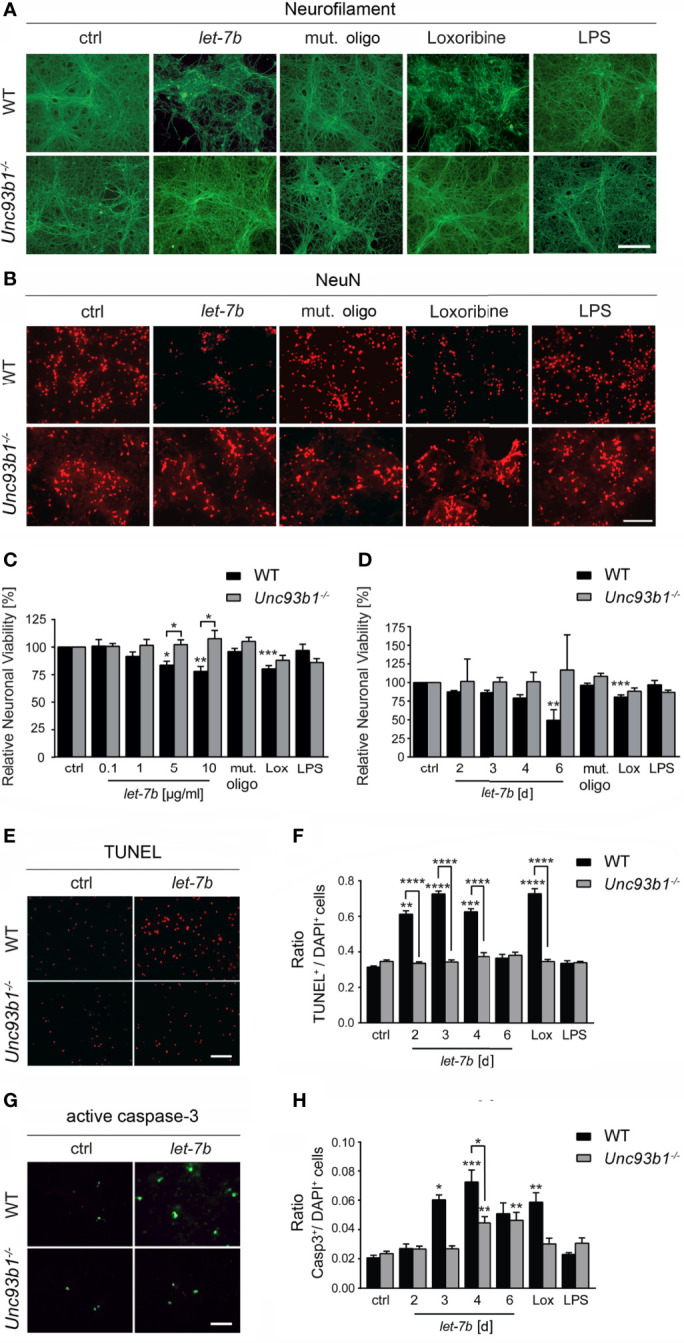
UNC93B1 is required for *let-7b*-induced cell-autonomous neuronal apoptosis *in vitro*. **(A, B)** Enriched cortical neurons isolated from C57BL/6 (wild-type, WT) and *Unc93b1^-/-^* mice were exposed to 10 µg/ml *let-7b* for 72 h or were left untreated (control). Mutant oligoribonucleotide (5 µg/ml) served as negative control. LPS (100 ng/ml) was used to indicate functionally relevant microglia cell numbers in the enriched neuronal cell cultures. Loxoribine (10 mM) served as positive control for TLR7-dependent neuronal injury. Subsequently, cells were immunolabeled using Neurofilament **(A)** and NeuN **(B)** antibodies to assess axonal damage and neuronal viability, respectively. **(C, D)** Cell cultures derived from WT and *Unc93b1^-/-^* mice described above were incubated with various doses of *let-7b*, as indicated, for 72 h **(C)** or treated with 5 µg/ml *let-7b* for different time periods, as indicated **(D)**. **(E–H)** WT cortical neurons were incubated with *let-7b* (5 µg/ml) for various time periods, as indicated, and were subsequently analyzed by **(E)** TUNEL assay and **(G)** immunostaining using active caspase-3 antibody. DAPI staining marked all present cells. Subsequently, TUNEL-positive and caspase-3-positive cells were quantified and normalized against DAPI-positive cells (**F** and **H**, respectively). Mutant oligoribonucleotide served as negative control, while loxoribine and LPS were used as positive control. **(C–H)** At least three independent experiments were performed. Data are expressed as mean ± SEM. Kruskal-Wallis test was used to determine global significance over all conditions [**(C)** *p* = 0.0042 over WT and *p* = 0.9557 over *Unc93b1^-/-^*; **(D)** *p* = 0.0036 over WT and *p* = 0.9971 over *Unc93b1^-/-^*; **(F)** *p* < 0.0001 over WT and *p* = 0.3514 over *Unc93b1^-/-^*; **(H)** *p* = 0.0010 over WT and *p* = 0.0036 over *Unc93b1^-/-^*]. *P* values of relevant groups (indicated condition *vs.* unstimulated control, ctrl) were determined by Dunn’s multiple comparison test. *P* values between respective groups of WT *vs. Unc93b1^-/-^* (in brackets) were assessed by unpaired Student’s *t* test. *p < 0.05; **p < 0.01; ***p < 0.001; ****p < 0.0001. Scale bar, 50 µm.

Exposure of neurons to extracellular miRNAs can trigger cell-autonomous injury through neuronal TLR7, independently on the presence of microglia (5). Here, we aimed at assessing the role of UNC93B1 in cell-autonomous neuronal apoptosis triggered by extracellular *let-7b*. To this end, highly enriched cortical neurons isolated from both WT and *Unc93b1^-/-^* mice were incubated with various *let-7b* doses, as indicated, for 72 h (**Figures 5A–C**). In addition, neurons were exposed to 5 µg/ml *let-7b* for different time periods, as indicated (**Figures 5D**). Subsequently, neuronal cultures were immunolabeled using neurofilament and NeuN antibodies. Nuclei of naïve *Unc93b1^-/-^* neurons appeared larger, and this enlargement even increased when neurons were exposed to the different TLR agonists, including *let-7b*, as indicated. Also, neuronal density in the *Unc93b1^-/-^* cell cultures seemed consistently to be slightly decreased compared to WT cell cultures (**Figure 5B**). We observed considerable axonal damage (**Figure 5A**) and loss of whole neurons (**Figure 5B**) in WT cell cultures in response to *let-7b* treatment, and the extent of damage was comparable to that observed in neuronal cultures incubated with loxoribine (**Figures 5A–D**). The neurotoxic effects induced by *let-7b* were dose- (**Figure 5C**) and time- (**Figure 5D**) dependent. In contrast, axonal morphology appeared unaffected (**Figure 5A**), and neuronal numbers were unaltered (**Figures 5B–D**) in neuronal cultures lacking UNC93B1 compared to control condition during the whole observation period, even when *let-7b* doses as high as 10 µg/ml were applied (**Figures 5A–D**). In line with these results, TUNEL staining revealed a significant increase in apoptotic cells in enriched WT neuronal cultures exposed to *let-7b* compared to control condition, while *Unc93b1^-/-^* neuronal cell cultures did not show such an effect over the whole observation period (**Figures 5E, F**). Whereas exposure to *let-7b* led to caspase-3 activation in WT neurons after 3 d, *Unc93b1^-/-^* neurons were not affected within this time period (**Figures 5G, H**). However, numbers of active caspase-3-positive *Unc93b1^-/-^* cells increased after 4 d. Still, at this time point numbers of caspase-3-positive cells derived from WT were significantly increased compared to those derived from *Unc93b1^-/-^* mice (**Figure 5H**). In contrast to WT neurons, *Unc93b1^-/-^* neurons were not affected by loxoribine-induced neurotoxicity (**Figures 5A–H**). LPS treatment did not induce neurotoxic effects, neither in WT nor in *Unc93b1^-/-^* neuronal cultures (**Figures 5A–H**). Whereas exposure of WT neurons co-cultured with WT microglia, but not *Unc93b1^-/-^* microglia, to 10 µg/ml *let-7b* resulted in a 40.7% reduction in relative neuronal viability (see **Figure 4E**), incubation of WT cortical neurons alone, but not *Unc93b1^-/-^* neurons, with 5 µg/ml and 10 µg/ml *let-7b* resulted in a 16.4% and 22.0% reduction in relative neuronal viability, respectively (**Figure 5C**). These data indicate that *let-7b* induces injury in enriched neurons, in accordance with our previous studies on cell-autonomous *let-7b*-induced neurodegeneration (5), and this effect increased in the presence of microglia (relative neuronal viability: neurons, 10 µg/ml *let-7b* neurons + WT microglia, vs. enriched neurons, *p* = 0.0101, Student’s *t*-test). Relevant contamination of enriched cortical neuronal cell cultures with glia was ruled out previously (5). Thus, we conclude that neuronal injury induced by *let-7b* is cell-autonomous, but can be enhanced by microglia.

Taken together, UNC93B1 expression contributes to cell-autonomous neuronal injury and apoptosis induced by *let-7b*.

### UNC93B1 Is Required for *let-7b*-Induced Neurodegeneration *In Vivo*

**Figure 6:**
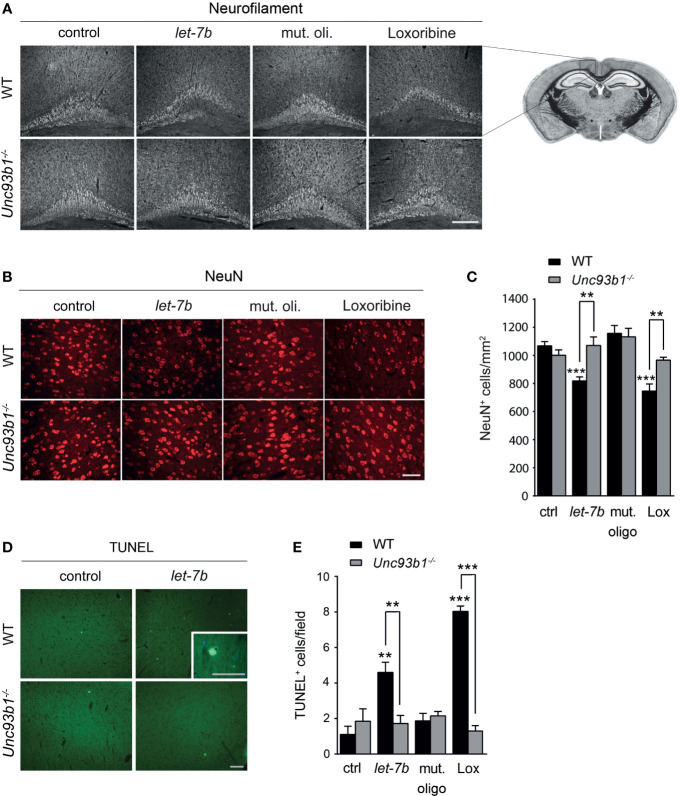
Neurodegeneration triggered by intrathecal *let-7b* and loxoribine requires UNC93B1 expression. We intrathecally injected 10 μg of *let-7b*, 10 μg of mutant oligoribonucleotide, 136 µg loxoribine, or PBS (control) into C57BL/6 (WT; let-7b, *n* = 7; mut. oligo, *n* = 6; loxoribine, *n* = 6; PBS, *n* = 4) or *Unc93b1^-/-^* (*let-7b*, *n* = 5; mut. oligo, *n* = 6; loxoribine *n* = 6; PBS, *n* = 4) mice. After 3 d, brain sections were immunolabeled with neurofilament **(A)** and NeuN **(B)** antibodies, and representative images of the pericallosal area and cerebral cortex, respectively, are shown [scale bar, 200 µm in **(A)**; 50 µm in **(B)**]. **(C)** Density of neurons in the cerebral cortex of WT and *Unc93b1^-/-^* mice was assessed quantifying NeuN-positive cells. Brain sections described above were stained with TUNEL assay **(D)**, and TUNEL-positive cells in the cerebral cortex were quantified **(E)**. **(C, E)** One-way ANOVA was used to determine statistical significances across all tested conditions **(C)** *p* < 0.0001 over WT and *p* = 0.1232 over *Unc93b1^-/-^*; **(E)** *p* < 0.0001 over WT and *p* = 0.571 over *Unc93b1^-/-^*]. Unpaired Student’s *t*-test was used to assess significant differences between pairs (indicated group *vs.* control, ctrl; WT *vs. Unc93b1^-/-^*; **p < 0.01; ***p < 0.001). Scale bar, 100 µm (overview **D**) and 50 µm (inlay in **D**).

To explore the role of UNC93B1 in *let-7b*- and TLR7-mediated neurodegeneration *in vivo*, WT and *Unc93b1^-/-^* mice were intrathecally injected with *let-7b* oligoribonucleotide, mutant oligoribonucleotide, or loxoribine. PBS served as negative control. After 72 h, brains were analyzed by immunohistochemistry using neurofilament and NeuN antibodies to mark axons and neurons, respectively. Injection of WT mice with *let-7b* resulted in lesions in the pericallosal area, and the extent of this damage was similar to that observed in mice treated with loxoribine (**Figure 6A**). *let-7b*-induced injury of the corpus callosum and diminished neurofilament immunolabeling was sequence-specific, as intrathecal mutant oligoribonucleotide did not induce such effects (**Figure 6A**). Quantification of NeuN-positive cells revealed significant loss of neurons in the cerebral cortex of WT mice treated either with *let-7b* or loxoribine, but not with mutant oligoribonucleotide, as expected. In contrast to WT mice, *Unc93b1^-/-^* mice were not affected by *let-7b-* or loxoribine-induced neurotoxicity. In detail, morphology of the corpus callosum and axons, as well as neuronal numbers in the cerebral cortex of *Unc93b1^-/-^* mice, did not differ between control, mutant oligoribonucleotide, loxoribine, and *let-7b* conditions (**Figures 6A–C**). In line with these findings, TUNEL staining of the cerebral cortex from WT mice injected with *let-7b* or loxoribine showed an increase in numbers of apoptotic cells, but did not reveal such effects in *Unc93b1^-/-^* mice (**Figures 6D, E**). Thus, we conclude that functional UNC93B1 expression is crucial for *let-7b*- and TLR7-triggered neurodegeneration *in vivo*.

## Discussion

TLRs and associated signaling pathways contribute to both injurious and regenerative processes in CNS diseases (33–35). We found recently that miRNAs can serve as signals for activation of TLRs in CNS cells, a function that is independent of their conventional role in post-transcriptional gene regulation. Specifically, *let-7* miRNAs are direct activators of TLR7 in both microglia and neurons (5). Subsequent studies validated our initial hypothesis that endogenous miRNAs, such as *let-7b*, are released from injured neurons, stimulate TLR7, thereby sending a danger signal to neighboring neurons, and cause further spread of CNS damage, particularly in the setting of neurodegenerative diseases (5, 15). However, the exact signaling cascades and molecular events linking TLR7 with microglia-mediated inflammation and neuronal apoptosis remained unresolved. Expression and function of the TLR trafficking protein UNC93B1 in the setting of human herpes encephalitis was previously described (13). Yet, the extent and cellular pattern of UNC93B1 expression in the brain, as well as this protein’s mode of regulation and functional impact on brain cells was unknown. Our data show that UNC93B1 is expressed in various CNS cells, including neurons, microglia, astrocytes, and oligodendrocytes. Moreover, it was required for microglial activation and neuronal injury through distinct TLRs, particularly for the response to extracellular *let-7b* miRNA, but also loxoribine, both presenting selective TLR7 activators. These results are in line with our previous findings on neurodegeneration triggered through TLR7 (5, 15), one of the client receptors for UNC93B1. Also, our findings are in accordance with previous studies, in which UNC93B1 expression was required for the microglial TNF-α response to whole bacteria and RNA (36) and for apoptosis of HEK293T cells (37).

We observed an increase in UNC93B1 expression in the developing mouse brain, while the expression of IRAK-1, representing a canonical TLR signaling-associated molecule, remained unchanged. Similar changes in mRNA expression of specific TLR family members, especially those linked to UNC93B1 function such as TLR3, TLR7, and TLR9, during mouse brain development have been previously described (38). Also, in accordance with our findings, Matcovitch-Natan and colleagues’ previous RNA-Seq studies indicate a steady increase in *Unc93b1* expression in murine microglia during the different brain developmental stages, reaching a peak at early postnatal stages (39). Functions of UNC93B1 beyond TLR trafficking and stabilization are poorly known (40), and so far, a role for this chaperone in a developmental setting has not been discussed. However, the findings that expression of UNC93B1 and specific TLRs changes during different developmental stages and that expression of other TLRs, TLR adaptors such as myeloid differentiation primary response 88 (MyD88), and associated downstream signaling molecules is low and unaltered at the same time (38), points to a, yet undetermined, role of the TLR chaperone and some TLRs in brain development. Notably, mice lacking UNC93B1 are viable, and although their brains have not been investigated at micro anatomic level so far, no major impairment in CNS development resulting in morphogenetic failures in these animals has been observed (8, 10) (data not shown). Thus, UNC93B1 may not be irreplaceable during brain development, and further molecules involved in TLR trafficking/stabilization and signaling may have to be considered when discussing the pathophysiological significance of TLR-linked chaperones in developmental processes in the vertebrate brain. Especially in the context of neurogenesis, select TLRs including TLR7, 8 and, 9 and associated signaling molecules have been suggested to play a functional role in brain development (38, 41–44). In line with this, our current data show that UNC93B1 is widely expressed in the CNS, not only in glial cell populations, but also in neurons. Neurons of various brain regions, in particular neocortex and hippocampus, express both TLR7 and TLR9, the interaction partners for UNC93B1 (5, 38). Further research will be needed to assess the functional impact of neuronal injury induced by miRNAs such as *let-7b* through UNC93B1, as observed in our current study, on vertebrate brain development. Also, our data indicate that UNC93B1 expression levels vary within a given CNS cell population, e.g. murine microglia, astrocytes, and neurons (see **Figure 1E**). UNC93B1 functions beyond TLR trafficking and stabilization are poorly known in general, even unexplored in CNS cells. Function and regulation of UNC93B1 expression may depend not only on the developmental stage, specific physiological and pathological conditions, or technical aspects, e.g. cell culturing, cell isolation procedures, potentially affecting one fraction within a cell population, but not the other one. Future studies may shed light on the potentially complex function of UNC93B1 in (individual) CNS cells. Finally, UNC93B1 function has been demonstrated to be altered by host and viral proteases targeting the chaperone’s N-terminus in HEK293T cells (37). Although it is conceivable that similar mechanisms exist in CNS cells, further investigation is required to decipher the potentially different functions of UNC93B1 in the vertebrate brain.

We found that UNC93B1 expression in neurons and microglia is upregulated not only by *let-7b*, but also by poly(I:C) and LPS, the established agonists for TLR3 and TLR4, respectively. This may point to a translational regulation with TLR7 agonists. Interaction between UNC93B1 and TLR3 in microglia was not surprising, as endosomal TLRs interact with UNC93B1 in immune cells such as dendritic cells and macrophages (8). The increase in UNC93B1 expression in response to LPS might result from activation of the TNF/interferon response factor/AP-1 signaling cascade, which is similar among all TLRs, subsequently leading to the transcription of UNC93B1. A direct interaction of UNC93B1 with the cell surface-localized TLR4 was not observed so far (7, 8, 10). Surprisingly, activation of TLR9, one of the UNC93B1 client receptors in peripheral immune cells, did not significantly modulate UNC93B1 expression, neither in microglia nor in neurons. Likewise, and in contrast to the observed effects of the miRNA ligand *let-7b* described above, loxoribine, a guanosine analog also known as a selective TLR7 agonist, failed to induce UNC93B1 expression in both cell types. Thus, regulation of UNC93B1 expression and function may be organ- and cell type-specific, but also dependent on the specific ligand activating the respective TLR. In turn, regulation of UNC93B1 expression may be crucial for modulating UNC93B1-dependent TLR function in brain cells, as it is seen in peripheral immune cells (11). In particular, elevated UNC93B1 expression was found to stabilize NA-sensing TLRs and render them hyperactive. Studies in models of systemic lupus erythematodes indicate that dysregulation of UNC93B1 might contribute to autoimmune processes by enhancing the expression of UNC93B1-dependent TLRs (45, 46). Also, competition between TLR7 and TLR9 for UNC93B1-mediated trafficking seems to be important for keeping NA-sensing TLRs in control of an inflammatory environment (47, 48). However, further studies are needed to determine whether TLR7 and TLR9 in microglia and neurons (5, 31) underlie such mechanisms. Also, the molecular structures responsible for the interaction between a given TLR and UNC93B1 in CNS cells, as they were defined as specific acidic amino acids in the juxtamembrane region of the receptors in peripheral immune cells (8), remain unresolved at this stage.

As described above, NA-sensing TLRs in peripheral immune cells depend on UNC93B1 to exit the ER (9), and similar conditions likely exist in CNS cells, particularly in microglia, the primary immune cells in the brain. Still, the function of UNC93B1 in endosomal trafficking, TLR protein stabilization, and prevention of their degradation in the different CNS cell types, as it was described in peripheral immune cells (11), is unknown. The clinical relevance of UNC93B1 expression in the brain lies in the observation that UNC93B1 deficiency in humans predisposes individuals to Herpes simplex virus 1 (HSV-1) encephalitis (13, 49), a life-threatening CNS infection causing severe neurological damage. This phenotype is suggested to result from impaired TLR3 signaling in neurons and oligodendrocytes (49). Consistent with this, an increased risk of herpes encephalitis after HSV-1 infection is observed in children with autosomal dominant mutations in TLR3, indicating that TLR3 trafficking by UNC93B1 is crucial for the control of HSV-1 infection (50). In addition, the fundamental function of UNC93B1 in maintaining TLR protein expression might be important in this context. The inability of *let-7b* and loxoribine to induce neuronal injury in UNC93B1-deficient mice as observed in our study might be caused, at least in part, by reduced/altered TLR expression in these mice. However, analyzing UNC93B1-deficient dendritic cells Brinkmann *et al.* had previously demonstrated that UNC93B1 deficiency does not affect TLR7 expression (10). Accordingly, our immunohistochemistry studies did not reveal significant differences in TLR7 expression or localization in the cerebral cortex between the two genotypes. We concluded that abolished neurotoxic effects in UNC93B1-deficient mice injected with TLR7 agonists are unlikely to result from altered TLR7 expression in these animals. However, we cannot rule out that the observed protection against the neurotoxic effects in UNC93B1-deficient mice injected with the TLR7 activators are due to an at least increased resistance towards neurotoxicity in general, i.e. alteration of non-TLR-specific apoptotic signaling pathways, in these mice. Future studies are required to shed light on these issues and may clarify whether TLRs and their interaction with UNC93B1 in different CNS cell populations are involved in the pathogenesis of herpes encephalitis and/or other infectious, but also non-infectious, CNS disorders associated with neuronal injury.

In summary, UNC93B1 is broadly expressed in the CNS. Our findings support a role for UNC93B1 in CNS development, inflammation, and injury, particularly triggered by extracellular miRNAs such as *let-7b*, which can serve as endogenous TLR7 activators. The determinants of UNC93B1 specificity for TLRs in CNS cells, as well as the molecular mechanisms by which UNC93B1 potentially mediates differential trafficking pathways and protein stabilization in the different cell types remain to be elucidated in future studies. These lines of research may not only contribute to our understanding of basic cell biological processes in brain cells, but may also provide deeper insight into the molecular mechanisms underlying various CNS diseases. Although efforts are underway to develop TLR inhibitors to treat brain disorders, especially autoimmune CNS diseases, those have primarily focused on the inhibition of ligand binding so far. Deciphering the pathways controlling TLR trafficking and localization in the brain, such as the ones involving UNC93B1, may lead to the development of novel therapeutic strategies.

## Data Availability Statement

Publicly available datasets were analyzed in this study. This data can be found here: http://www.brainrnaseq.org/.

## Ethics Statement

The animal study was reviewed and approved by Landesamt für Gesundheit und Soziales – LAGeSo, Berlin, Germany.

## Author Contributions

SL and MB conceived the study and wrote the manuscript. MK, OD, TW, CK, DG, AB, KD, and HK planned and/or carried out the experiments. All authors contributed to the article and approved the submitted version.

## Funding

This work was supported by NeuroCure Exc 257, Deutsche Forschungsgemeinschaft (DFG; LE 2420/2-1, SFB-TRR167/B03, to SL), the Helmholtz Association (W2/W3-090 to MMB), Berliner Krebsgesellschaft, Monika Kutzner Foundation, and Else Kroener-Fresenius Foundation (to OD).

## Conflict of Interest

The authors declare that the research was conducted in the absence of any commercial or financial relationships that could be construed as a potential conflict of interest.

## Publisher’s Note

All claims expressed in this article are solely those of the authors and do not necessarily represent those of their affiliated organizations, or those of the publisher, the editors and the reviewers. Any product that may be evaluated in this article, or claim that may be made by its manufacturer, is not guaranteed or endorsed by the publisher.
