# Supplementary material for: UNC93B1 Is Widely Expressed in the Murine CNS and Is Required for Neuroinflammation and Neuronal Injury Induced by MicroRNA let-7b
Source: Front Immunol. 2021 Sep 13;12:715774. doi: 10.3389/fimmu.2021.715774 (PMC8475950; doi:10.3389/fimmu.2021.715774)
Supplement: Supplementary file 1 [file DataSheet_1.pdf]

Supplementary Material

Species	Cell type/cell stage	UNC93B1 expression	
Mouse	Astrocytes	1.99	Zhang et al. 2014
	Neurons	0.99	
	Oligodendrocyte precursor cells	20.43	
	Newly formed oligodendrocytes	5.06	
	Myelinateing oligodendrocytes	14.13	
	Microglia/macrophage	582.26	
	Endothelial	8.49	
Mouse	Microglia E17	178.15	Bennett et al. 2016
	Microglia P7	404.46	
	Microglia P14	494.27	
	Microglia P21	356.61	
	Microglia P60	318.80	
	Microglia/macrophage	1,119.25	
Human	Fetal astrocytes	0.10	Zhang et al. 2016
	Mature astrocytes	0.24	
	Neurons	0.10	
	Oligodendrocytes	0.10	
	Microglia/macrophage	0.44	
	Endothelial	0.12	

Supplementary Figure 1. RNA-Seq analysis of *Unc93b1* expression in different brain cell populations from mouse and human. *Unc93b1* expression is provided as fragments per kilobase per million (FPKM). Red bars represent expression values. Data was extracted from datasets published in Zhang et al., 2014, Zhang et al., 2016, and Bennett et al. 2016, available at https://www.brainrnaseq.org/.

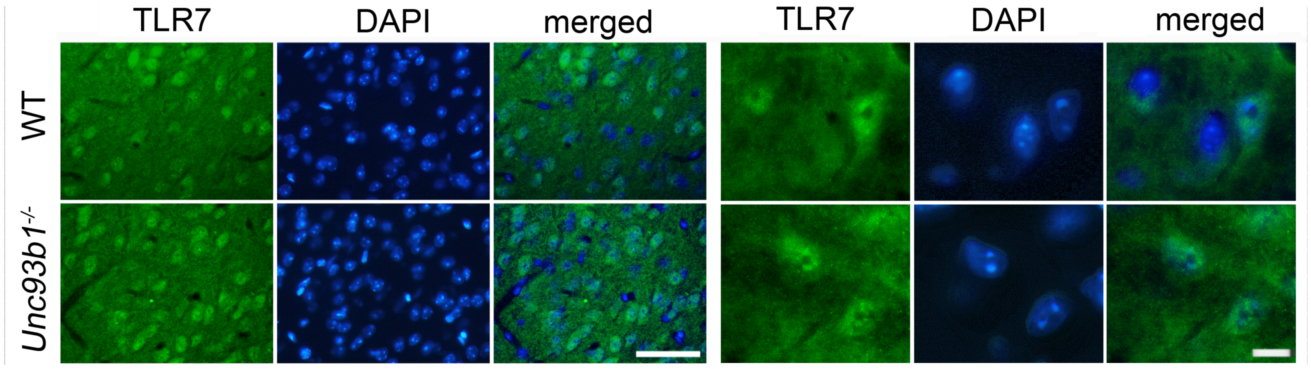

Supplementary Figure 2. TLR7 expression in cerebral cortex of C57BL/6 and *Unc93b1*$^{-/-}$ mice. Brain sections from WT (*n* = 4) and *Unc93b1*$^{-/-}$ (*n* = 4) mice were immunolabeled using an antibody directed against TLR7. An overview (left panel; scale bar, 50 μm) and higher magnification images (right panel; scale bar, 10 μm) of the cerebral cortex displaying TLR7 expression are shown.

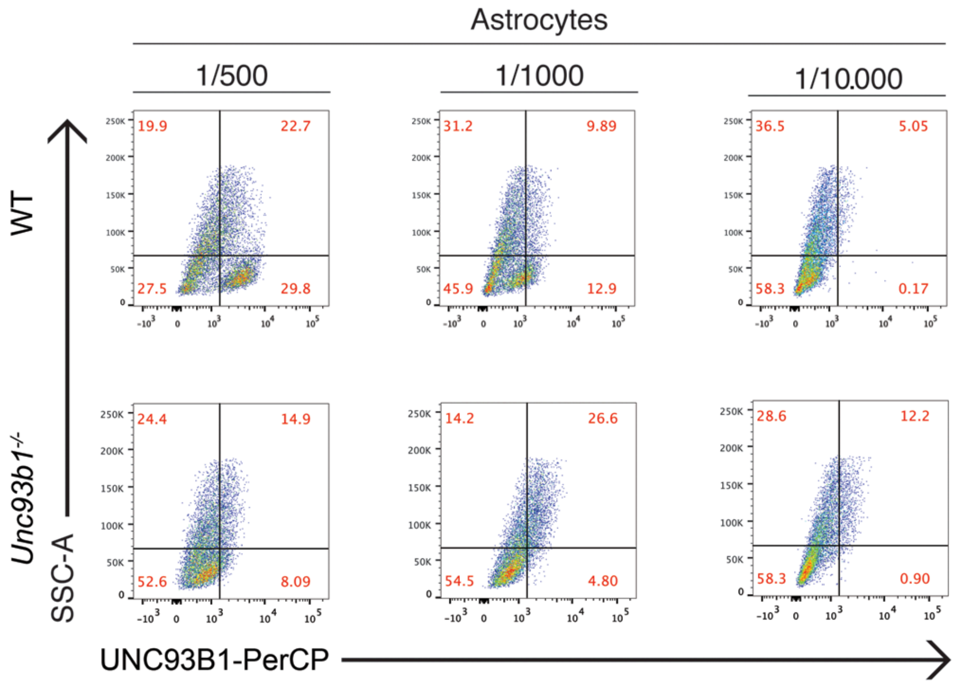

Supplementary Figure 3. Validation and titration of the UNC93B1-N antibody used for flow cytometry. Astrocytes isolated from C57BL/6 and *Unc93b1*$^{-/-}$ mice were stained with UNC93B1-N antibody generated in M. Brinkmann's laboratory, using different antibody concentrations, as indicated. Subsequently, cells were analyzed by flow cytometry. One representative experiment out of 3 with similar results is shown.
